# Real-time *in Vivo* monitoring of photosynthesis in individual leaves by frequency-locked quartz-enhanced photoacoustic spectroscopy

**DOI:** 10.1016/j.pacs.2026.100802

**Published:** 2026-01-23

**Authors:** Surui Liu, Lu Qin, Chongqiu Zhou, Juncheng Lu, Wen Liu, Jie Shao

**Affiliations:** Department of Optical Engineering, School of Physics and Electronic Engineering, Zhejiang Normal University, Jinhua 321004, China

**Keywords:** Quartz-enhanced photoacoustic spectroscopy, 1f-locking technique, CO2 gas sensor, Photosynthetic rate, Rapid measurement

## Abstract

This paper presents a method for real-time measurement of *in vivo* leaves photosynthetic rates using quartz-enhanced photoacoustic spectroscopy (QEPAS) with first-harmonic frequency- locking (1f-locking). A compact gas sensor structure was constructed by integrating an in-plane acoustic micro-resonator (AmR) with a commercial 30.720 kHz quartz tuning fork (QTF). The DFB laser’s output wavelength was locked to the CO_2_ absorption line at 4991.26 cm^−1^ via 1f-locking, enabling real-time monitoring of CO_2_ uptake during leaf photosynthesis. Compared to the scanning mode, the standard deviation (STD) in 1f-locking mode is significantly reduced, with detection sensitivity increased by nearly threefold. The system achieved a 1 s measurement cycle, with detection linearity R^2^ = 0.999. When the integration time is 127 s, the minimum detection limit (MDL) is 2.44 ppmv. The normalized noise equivalent absorption coefficient (NNEA) is 4.78 × 10^−9^ cm^−1^·W·Hz^−1/2^. Results obtained align with reported photosynthetic rate ranges, validating the system’s feasibility. This system provides a portable, highly sensitive, rapid, and reliable method for leaves photosynthetic rate determination.

## 1 Introduction

Under the “dual-carbon” target, boosting carbon sequestration has become a key part of climate action [1], [2], [3], [4]. Photosynthesis is the fundamental process by which plants convert light energy into organic matter and is a core link in the energy transfer and carbon cycle of ecosystems. The measurement of photosynthesis is not only a key means for understanding the state of plants and breeding high photosynthetic efficiency crops but also an important scientific basis for responding to climate change and enhancing the service functions of ecosystems. Leaves fix atmospheric CO_2_ through photosynthesis, providing a direct and renewable carbon sink [5], [6]. To exploit this potential, the CO_2_ uptake rate of a single leaf must first be known: only with data from the same genotype at different growth stages or under different stresses can high-efficiency germplasm be selected and management practices optimized, allowing up-scaled predictions of ecosystem carbon flux [7].

Compared with eddy-covariance towers or satellite observations, single-leaf measurements in controlled conditions exclude soil respiration and turbulent mixing and yield a photosynthetic rate set purely by stomatal conductance and carboxylation capacity, forming the physiological link between gene, phenotype, and carbon budget [8], [9], [10]. As the primary site of photosynthesis, leaves provide the strongest, most stable, and easily standardized gas exchange signals, making them the preferred unit of measurement in most photosynthesis studies. Accurate quantification of the resulting ppmv CO_2_ depletion inside a rapidly humidifying leaf chamber, however, demands a gas-analytical technique whose selectivity and stability exceed the limits of conventional sensors, thereby motivating the adoption of optical absorption strategies [11], [12]

Among optical methods, absorption spectroscopy is widely used because of its selectivity and speed. Non-dispersive infrared (NDIR) sensors are cheap and mature, but their broadband filter cannot separate CO_2_ from overlapping H_2_O bands, and the signal drifts with temperature and humidity. In a closed leaf chamber, relative humidity can reach 90 %, and a ± 2 °C fluctuation can introduce tens of ppmv of apparent CO_2_ change, far above the few-ppmv draw-down caused by a leaf photosynthesizing at μmol·m^−2^·s^−1^ levels [13], [14], [15].

Another optical method is based on tunable diode laser spectroscopy (TDLAS), which utilizes lasers with a narrow line width and can scan a single specific absorption line of CO_2_ molecules. It can exclude interferences from other exhaled gases and provide very precise measurement, especially after the combination with wavelength modulated spectroscopy (WMS) [16], [17], [18], [19], [20]. In 2019, Lou et al. developed a TDLAS-based system for online detection of human exhaled CO_2_, achieving a minimum detection limit of 0.769 % and a response time of approximately 10 ms, providing a new strategy for non-invasive detection of exhaled gases and related disease markers [21]. However, the bulk sensor was cumbersome due to its weight, and the mid-infrared photon detector (PD) was costly and susceptible to environmental noises [22].

QEPAS is an optimized technique developed from traditional photoacoustic spectroscopy (PAS) [23], [24], [25], [26], [27], [28], [29]. In 2002, QEPAS was proposed for the first time and has been popular ever since [30]. The QTF, as a cheap mini piezoelectric crystal in clocks with a high Q factor, is used to detect sounds induced by light absorptions of gas molecules [31], [32]. In recent years, QTF have been employed not only in QEPAS but also as cantilever sensors for gas detection [33]. Researchers have enhanced the detection sensitivity of QEPAS by designing customized low-frequency QTFs [34], constructing different acoustic resonance detection structures [35], [36], [37], using high-power lasers [38], and conducting multiple excitations [39]. QEPAS technology features high sensitivity, excellent selectivity, a compact detection structure, low cost, and strong suppression of 1/*f* noise [38], enabling applications in environmental monitoring, medical diagnostics, and industrial process measurement [40], [41], [42], [43], [44], [45]. In 2019, Wu et al. used QEPAS to obtain a MDL of 3.5 ppmv in CO_2_ monitoring [46]. In 2020, Nicolas et al. obtained a MDL of 20 ppbv in 1 s with a QEPAS CO sensor and applied it in the clinic for human breath analysis [47]. In 2023, Zhang et al. established a QEPAS-based CO_2_ sensor with a MDL of 2.6 ppmv and a response time of 3 s to investigate the emitted CO_2_ of human skin [48].

Unfortunately, conventional QEPAS acquires the full absorption profile by sweeping the laser, and a complete QEPAS signal acquisition cycle typically requires ∼10 s, too slow to follow the rapid CO_2_ drop during the first minute of leaf photosynthesis. 1f-locking fixes the laser wavelength at the absorption peak and uses the first-harmonic signal as an error signal to correct frequency drift, reducing the measurement time to ≤ 1 s while maintaining peak absorption strength. The 1f-locking technique modulates the laser frequency and detects the first-harmonic component of the absorbed signal to achieve feedback control of the laser frequency deviation from the absorption line center [49]. This enables the laser to be stably locked at the absorption peak position, which can significantly reduce the detection time of the measurement system.

In this work, 1f-locking QEPAS is coupled with an in-plane acoustic micro-resonator and targeted at the H_2_O-free CO_2_ line at 2004 nm (4991.26 cm⁻¹) to realize real-time photosynthesis monitoring inside a closed leaf chamber. A traditional closed system was selected to achieve high temporal resolution and non-continuous dynamic monitoring of the CO_2_ absorption process in plants due to its simple device, convenient operation, and rapid measurement. The system updates at 1 s, achieves the MDL of 2.44 ppmv, and has linearity R^2^ = 0.999, offering a sensitive, humidity-immune and fast tool for single-leaf CO_2_ flux measurements. The combined application of spectrum-line-optimized QEPAS technology and 1f-locking techniques offers an effective approach to address gas detection challenges in high-humidity, enclosed environments, holding promise for achieving in-situ, real-time, and precise monitoring of plant photosynthesis.

## 2 Theory

To realize the real-time monitoring, the gas detection in this work was based on a wavelength stabilized technique. Here is a detailed explanation of the theory behind this technique. According to the Beer-Lambert law, the transmitted light intensity can be expressed as:

(1) $$I\left(t\right)=I_{0}(t)\tau \left[v\left(t\right)\right]=I_{0}(t)\exp(-\alpha \left[v(t)\right]l)$$

where I is the transmitted light intensity, v is the light frequency, $\tau \left[v\left(t\right)\right]$ is the transmission coefficient, $I_{0}$ is the incident light intensity, $\alpha \left[v(t)\right]$ is the absorption coefficient, l is the optical length of the absorbing medium.

In WMS, the incident light is modulated by a sinusoidal waveform and the instantaneous frequency can be expressed as:

(2) $$v\left(t\right)=\overset{®}{v}+∆v\cos(2\pi ft)$$

where v is the instantaneous light frequency, $\overset{®}{v}$ is the center frequency of the absorption line, ∆v is the modulation amplitude, f is the modulation frequency that is half of the resonant frequency ($f_{0}$) of the QTF.

Thus, the transmission coefficient can be expressed as:

(3) $$\tau \left[v\left(t\right)\right]=\sum_{k=0}^{+\infty }H_{k}\left(\overset{®}{v},∆v\right)\cos\left(2\pi \mathrm{kft}\right)$$

where $H_{k}\left(\overset{®}{v}\right)$ are given by [50]:

(4) $$H_{0}\left(\overset{®}{v},∆v\right)=\frac{1}{2\pi }\int_{-\pi }^{+\pi }\tau \left[\overset{®}{v}+∆v\cos(2\pi \mathrm{ft})\right]\mathrm{du}$$

(5) $$H_{k}\left(\overset{®}{v},∆v\right)=\frac{1}{\pi }\int_{-\pi }^{+\pi }\tau \left[\overset{®}{v}+∆v\cos(2\pi \mathrm{ft})\right]\mathrm{coskudu}$$

According to the spectral line-shape theory of WMS, the harmonic components of the modulated absorption signal exhibit distinct symmetry characteristics. Under ideal conditions, the first-harmonic (1 f) signal presents an antisymmetric profile with an intrinsic zero-crossing feature in the vicinity of the absorption line center, whereas the second-harmonic (2 f) signal exhibits a symmetric line shape and reaches its maximum amplitude at the line center [51]. Owing to these properties, the 1 f signal is commonly employed as an error signal for laser wavelength locking, while the amplitude of the 2 f signal is primarily utilized for gas concentration retrieval. In practice, both harmonics are retrieved with a lock-in amplifier.

Although the 2 f signal is widely used for concentration measurement in gas sensing applications, its symmetrical linear characteristics alone do not inherently provide the zero-crossing feature required for frequency stability. When applied to wavelength locking, additional signal processing strategies such as slope-based locking or phase optimization are typically needed, which may increase the complexity of the feedback control loop. In contrast, the inherent zero-crossing characteristics of the 1 f signal enable direct and unambiguous wavelength locking, making it particularly suited for robust frequency stabilization.

**Fig. 1:**
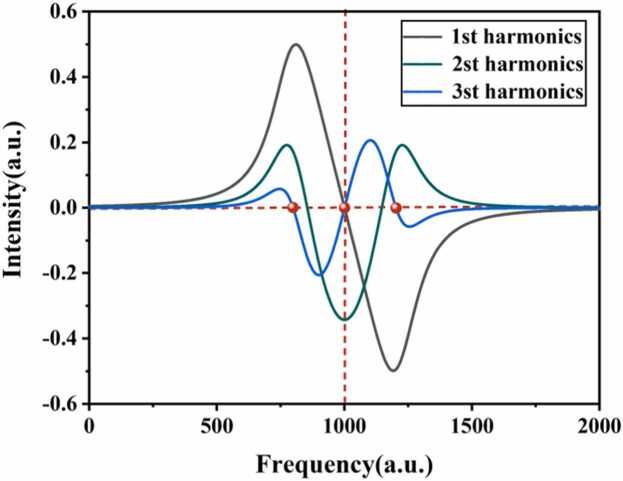
Simulated waveform diagrams for each harmonic.

Higher-order harmonic locking techniques, such as third-harmonic (3 f) locking, have also been reported in high-stability spectroscopic systems. However, as shown in Fig. 1, the 3 f signal exhibits a significantly lower amplitude compared to the 1 f signal and features multiple zero crossings near the absorption line center. Especially under low gas concentration and low signal-to-noise ratio conditions, these multiple zero-crossing features may increase the risk of false locking, thereby degrading system stability. Therefore, considering signal amplitude, zero-crossing uniqueness, and overall system robustness, the 1 f signal is selected as the preferred error signal for wavelength locking in this work.

In real gas sensing systems, background absorption, optical interference, and residual amplitude modulation are unavoidable, so a non-zero 1 f signal outside the target absorption line is a common phenomenon. However, as long as the background remains stable, its effect mainly introduces a constant offset in the zero-crossing position and does not degrade the stability of frequency locking [52].

## 3 Experimental system

### 3.1 Selection of CO_2_ absorption lines

**Fig. 2:**
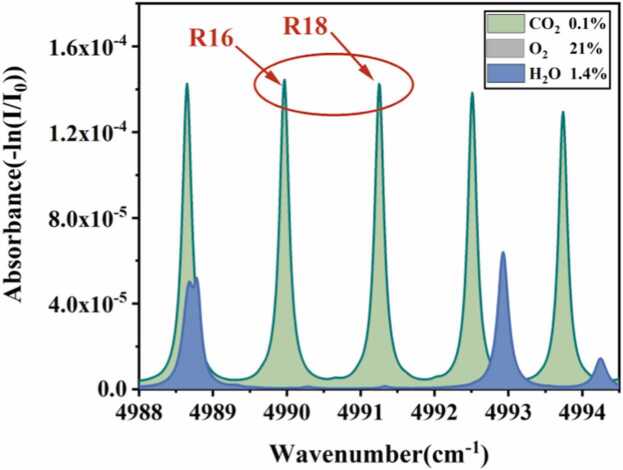
Simulation of absorption lines for major photosynthetic gas components based on the HITRAN 2020 database.

Since plant photosynthesis directly involves processes such as CO_2_ uptake, O_2_ release, and H_2_O transpiration, it is essential to eliminate interference from changes in O_2_ and H_2_O concentrations for accurate detection of CO_2_ concentration variations during photosynthesis. This experiment employed a continuous-wave distributed feedback laser (CW-DFB, Nanoplus, 2004 nm) as the excitation source, targeting the CO_2_ absorption line near 4991.26 cm^−1^. Based on the HITRAN 2020 database, the absorption spectra of various gases in air (21 % O_2_, 1.4 % H_2_O, 0.04 % CO_2_) were simulated in the 4988–4995 cm^−1^ range, as shown in Fig. 2. Spectral lines R16 and R18 exhibit similar line intensities, but R18 is farther from the H_2_O absorption line. To avoid interference from water vapor on CO_2_ gas detection, we selected spectral line R18 for all subsequent experiments.

### 3.2 Detection structure

**Fig. 3:**
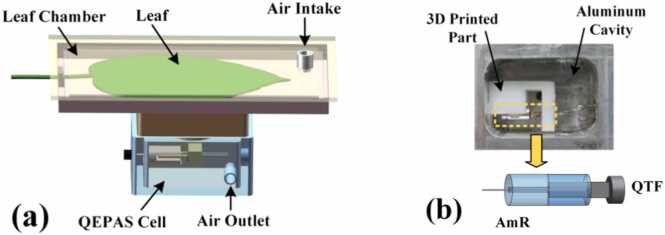
Components of the gas detection structure. (a) 3D schematic diagram of the gas detection structure based on QEPAS; (b) Physical photograph of the QEPAS module and 3D schematic diagram of the in-plane incident structure. AmR: acoustic micro-resonator; QTF: quartz tuning fork.

As shown in Fig. 3(a), the detection structure consists of two parts: the leaf chamber and the QEPAS cell. The leaf chamber was constructed with a transparent, removable acrylic box structure for fixing and positioning leaves during photosynthesis detection. The structure of the QEPAS cell is shown in Fig. 3(b). It employed an in-plane excitation configuration and consisted of an aluminum cell (inner dimensions: 2.6 cm × 1.8 cm × 1.4 cm), 3D-printed support components, an AmR (OD = 3.2 cm, ID = 0.5 cm), and a QTF (f = 30.704 kHz). The AmR dimensions adopted by this research institute follow the systematic optimization results previously established by our team for different lengths and inner diameters [37]. This structural design enables efficient excitation and detection of acoustic signals within a limited volume, while achieving stable alignment between optical paths and acoustic components through precision support structures. The integration of the leaf chamber and the QEPAS cell ensures efficient coupling of photoacoustic signals while facilitating compact integration.

### 3.3 The gas detection system

**Fig. 4:**
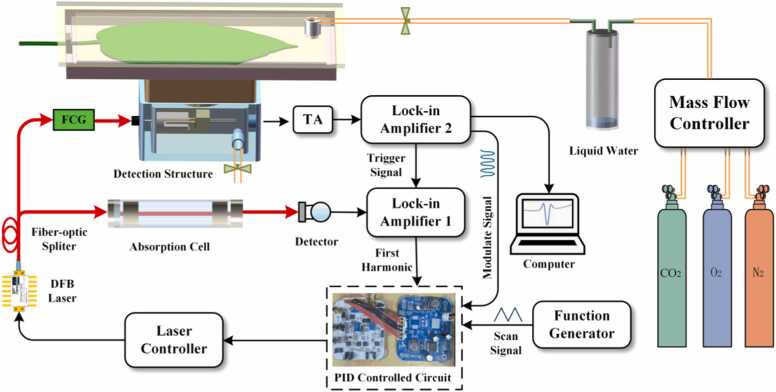
Schematic diagram of QEPAS combined with 1f-locking technology for the photosynthesis gas detection system. FCG: fiber-coupled Grin collimator; TA: transimpedance amplifier.

The experimental setup is shown in Fig. 4. During operation, the DFB was modulated by superimposing a triangular wave (150 mHz, 800 mV) and a sine wave (f₀/2 = 15.352 kHz) to achieve wavelength scanning and harmonic modulation. The modulated laser beam was split into two beams by a fiber optic splitter, with 5 % of the optical power allocated for frequency locking. After passing through an absorption cell filled with pure CO_2_, the beam was received by a photodetector and then fed into a lock-in amplifier (LIA1) for first-harmonic demodulation. 95 % of the laser beam incident in the gas detection structure was directed toward CO_2_ gas detection. After being shaped by a fiber-coupled Grin collimator (FCG, f = 10 mm), the laser was focused onto the QTF, causing it to induce symmetric vibrations. The piezoelectric effect based on QTF converts mechanical vibrations into electrical signals. The electrical signal output from the QTF was amplified by a preamplifier and then sent into another lock-in amplifier (LIA2) for secondary harmonic demodulation, thereby inverting the volume fraction of the CO_2_ to be measured. In this case, the reference signal for LIA1 was supplied externally by LIA2, ensuring phase synchronization of the demodulated signal. After stabilizing the acquisition of 1 f and 2 f signals, the system controls the laser wavelength via a PID controller. This causes the real-time acquired 1 f signal voltage to approach a preset reference voltage, thereby locking the laser frequency at the absorption line center. This reference voltage serves as the zero-error setpoint for the PID loop. To prove the feasibility of combining QEPAS with 1f-locking technology to rapidly measure plant leaf photosynthetic rates, a gas flow control module was incorporated into the experimental system. Considering that CO_2_ concentrations in indoor environments are easily affected by external disturbances, a gas delivery system was employed to provide stable and controllable CO_2_ concentrations during the initial experimental phase to ensure consistent initial conditions. In practical applications, this method enables in situ measurement without requiring additional gas flow systems.

## 4 Sensor performance analysis

### 4.1 QTF optimal frequency selection

**Fig. 5:**
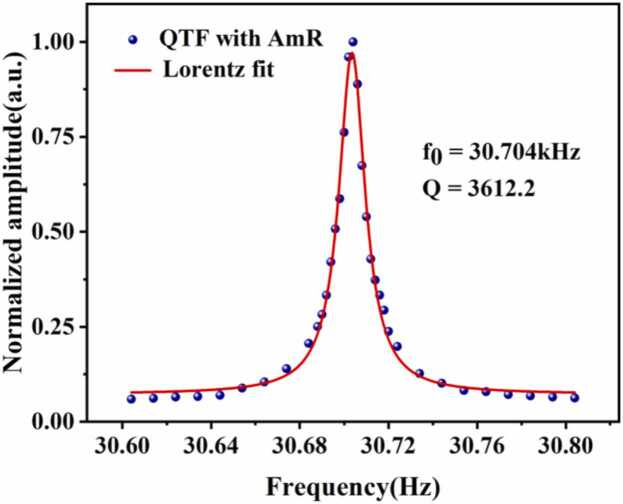
Frequency response curve of QEPAS ADM.

Since QEPAS technology incorporates modulation techniques, the resonant frequency of the QTF determines the modulation frequency of the excitation light. However, the resonant frequency of a QTF under actual atmospheric pressure often deviates from its labeled frequency, necessitating calibration of the QTF's resonant frequency. A commercially available quartz tuning fork with a resonant frequency of 30.720 kHz was used in the experiment. After assembling the QTF with the in-plane AmR, a frequency scan test was performed on the tuning fork. The amplitude output at each frequency was recorded to obtain the quartz tuning fork's frequency response curve. The test results are shown in Fig. 5, with f_0_ = 30.704 kHz, a bandwidth of 8.5 Hz, and a Q factor of 3612.2. The Q factor of QEPAS ADM is reduced compared to that of bare QTF (typically around 10,000), primarily attributed to enhanced internal energy transfer and storage within the AmR. This indicates good acoustic-energy coupling between the QTF and the AmR.

### 4.2 Frequency-locked signal

**Fig. 6:**
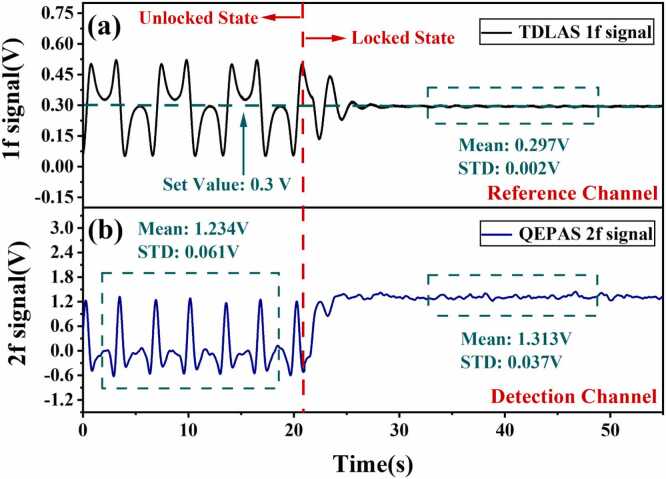
Frequency-locked signal. (a) TDLAS 1 f signal from unlocked state to locked state; (b) QEPAS 2 f signal from unlocked state to locked state.

The demodulated 1 f signal from the absorption cell was fed into the analog PID control circuit to serve as the error signal for PID frequency locking. The PID module iteratively adjusted the current offset to bring the 1 f signal closer to the set value, thereby locking the laser output frequency at 4991.26 cm^−1^. A standard gas at a fixed concentration was continuously introduced into the QEPAS cell. The dynamic response characteristics of the locking process were real-time reflected through synchronously monitored 1 f signals and 2 f signals, with the specific results shown in Fig. 6. Due to trigger synchronization, the 2 f and 1 f curves share the same horizontal axis. After determining the center position of the 2 f curve, the center setting value of 0.3 V for the absorption line corresponding to the synchronized 1 f curve can be confirmed. After stabilization, the average amplitude of the 1 f signal is 0.297 V (STD = 1.8 × 10^−3^ V), with a relative error of 1 % compared to the setpoint value. Fig. 6(b) displays the 2 f signal generated over three tuning cycles in the unlocked state (since the tuning signal is a triangular wave, six 2 f signals are generated). Due to the residual amplitude modulation (RAM) effect, the two sides of the 2 f signal at the target wavelength center exhibit asymmetry, with mean and STD values of 1.234 V and 0.061 V, respectively, representing a relative deviation of 4.94 %. In contrast, during the frequency-locked period, the mean and STD of the 2 f signal amplitude are 1.313 V and 0.037 V, respectively, producing a relative deviation of 2.82 %. This indicates that in the frequency-locked state, the relative deviation of the 2 f signal is reduced by 42.91 %. This improvement stems from two factors: first, by locking the laser frequency to the center of the absorption line, RAM interference with the concentration detection signal is effectively suppressed, thereby enhancing the system's long-term stability, and second, the locked state permits the use of a narrower phase-locked detection bandwidth, further suppressing broadband noise.

### 4.3 Impact of lock-in amplifier bandwidth in different modes

**Fig. 7:**
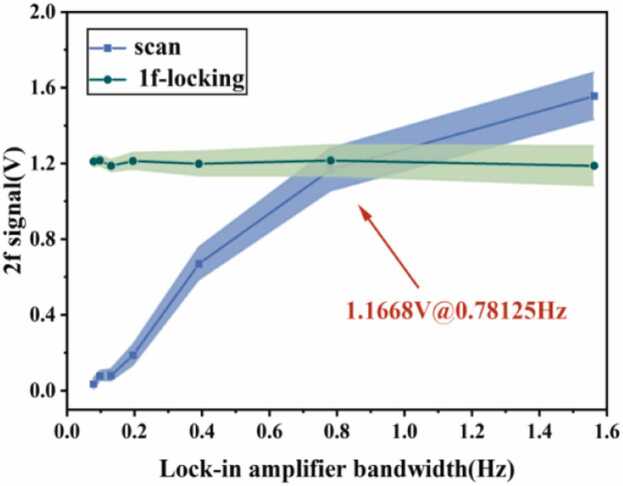
Photoacoustic signal corresponding to different lock-in amplifier bandwidths in scan mode and 1f-locking mode. The shaded area represents the error band.

In QEPAS systems, the equivalent noise bandwidth of the lock-in amplifier exhibits fundamental differences between two operating modes, as illustrated in Fig. 7. During scanning mode, the scanning frequency must precisely match the lock-in time constant to resolve absorption spectral lines. If the bandwidth is too narrow, it smooths or even distorts the absorption line shape, reducing resolution. Conversely, excessive bandwidth introduces too much noise, degrading the SNR. This trade-off complicates system parameter optimization. In contrast, frequency-locked mode fixes the excitation light at the center of the absorption peak, cleverly avoiding this conflict. Its core advantage lies in minimizing bandwidth, suppressing detector noise and external interference to theoretical limits. A standard gas at a fixed concentration was continuously introduced into the QEPAS cell. The measurement results obtained under different modes are shown in Fig. 7. In scan mode, with a bandwidth of 0.78 Hz, the STD is 0.1132 V. In frequency-locked mode, the bandwidth can be set to a minimum of 0.07813 Hz, yielding an STD of 0.0294 V. Compared to scan mode, STD decreased by 74 %, with detection sensitivity increasing nearly threefold. This design maximizes the system's SNR without altering signal strength, enabling long-term stable ultra-high-sensitivity detection. Consequently, frequency-locked mode achieves a qualitative leap in single-point detection performance, with its core advantages being exceptional long-term stability and sensitivity approaching theoretical limits.

### 4.4 CO_2_ gas calibration and Allan deviation

**Fig. 8:**
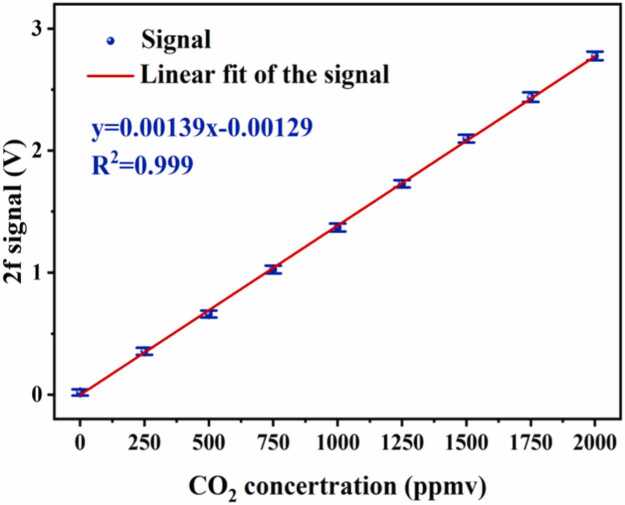
The linear relationship between CO_2_ concentration and 2 f signal peak values.

Based on the gas absorption principles of QEPAS, the photoacoustic signal amplitude exhibits a good linear relationship with the gas concentration. To evaluate the linear response of the gas detection system to CO_2_, we measured various concentrations of CO_2_ gas using the frequency-locked mode. A mass flow controller (MFC, CS200-A, Beijing Seven Star HuaChuang Electronics, accuracy ±1.0 %) was employed to configure different concentrations of CO_2_ gas. By mixing 2000 ppmv CO_2_ gas with high-purity N_2_ (Jinhua Datong Gas) at different flow rates, the following CO_2_ concentration gradients were obtained: 0 ppmv, 250 ppmv, 500 ppmv, 750 ppmv, 1000 ppmv, 1250 ppmv, 1500 ppmv, 1750 ppmv, and 2000 ppmv. At standard atmospheric pressure, the gas flow rate was controlled at 400 mL/min. The gas was sequentially introduced into the detection structure, and 200 sets of 2 f signals were collected. Linear fitting was performed on signals of different concentrations, with the vertical error bars representing the STD of the 200 signals, with results shown in Fig. 8. According to Fig. 6, the QEPAS signal increases with rising CO_2_ concentration. The linear fit resulted in a sensitivity of 1.39 × 10^−3^ V/ppmv and an R^2^ value of 0.999, indicating an excellent linear response of the QEPAS-based CO_2_ sensor system.

**Fig. 9:**
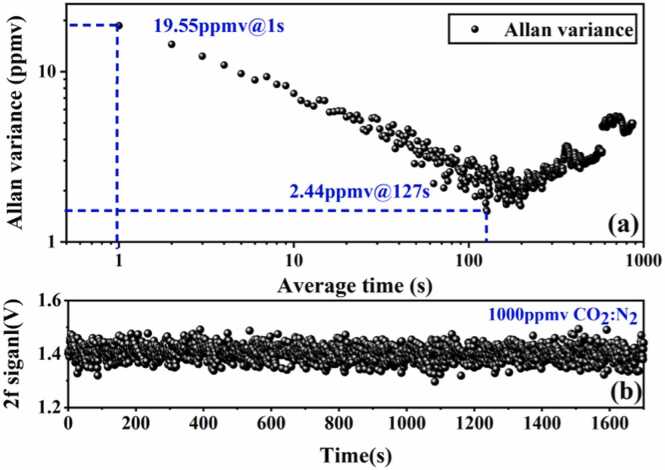
System stability test. (a) Allan variance analysis for the CO_2_ sensor; (b) 2 f signal within 30 min after locking.

Followed by, to test the long-term stability and detection limits of the CO_2_ sensor, a CO_2_-N_2_ gas mixture containing 1000 ppmv CO_2_ was continuously introduced into the detection structure. In frequency-locked state, the 2 f signal obtained from QTF response demodulation was continuously monitored for 30 min, with the experimental sampling rate set to 1 Hz, as shown in Fig. 9(b). To evaluate the system's long-term stability, Allan variance analysis was performed on the data in Fig. 9(b), with results shown in Fig. 9(a). At an integration time of 1 s, the MDL was 19.55 ppmv. At the optimal integration time of 127 s, the QEPAS system achieves the MDL of 2.44 ppmv. The normalized noise equivalent absorption coefficient (NNEA) of the system is calculated to be 4.78 × 10^−9^ cm^−1^·W·Hz^−1/2^.

### 4.5 Static system stability test

Before employing the CO_2_ sensor to detect photosynthetic rates, to ensure the measurement accuracy of CO_2_ concentration changes within the closed system and thereby precisely calculate plant photosynthetic rates, we first evaluated the gas leakage conditions of this system. To quantitatively assess the gas leakage rate of this closed system, we conducted a static stability test. The initial CO_2_ concentration for photosynthetic experiments was set at 1000 ppmv. This selection was based on the practical observation that indoor experimental environments typically exhibit CO_2_ levels higher than outdoor background values. This setting also aligns with the initial concentration levels commonly used in existing studies [52] measuring photosynthesis in closed systems. During the photosynthetic measurements of plant leaves in this experiment, the CO_2_ concentration within the chamber primarily fluctuated within the range of approximately 750–1000 ppmv.

**Fig. 10:**
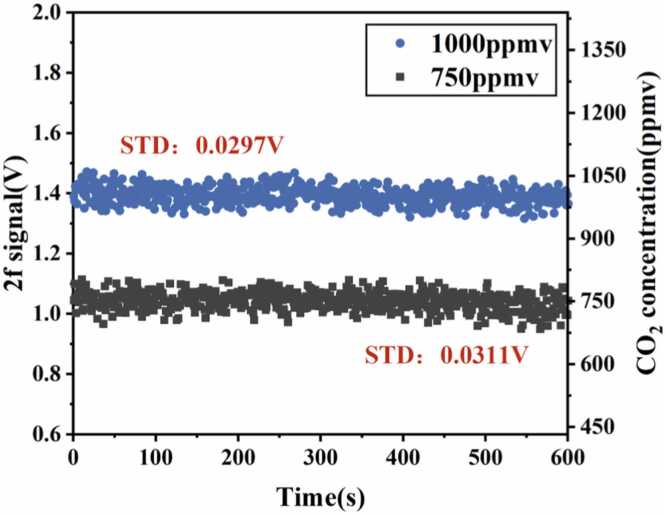
Stability test for gas detection structure at concentrations of 1000 ppmv and 750 ppmv.

Using an MFC, two concentrations were prepared. CO_2_ gas at concentrations of 1000 ppmv and 750 ppmv was introduced into the detection structure for two minutes each. Once the CO_2_ concentration within the detection structure stabilized, both the gas inlet and outlet ports were closed to create a sealed system. A 10-minute stability test was then performed on the detection structure. The results are shown in Fig. 10. At a concentration of 1000 ppmv, the STD of the signal is 2.97 × 10^−2^ V; at a concentration of 750 ppmv, the STD of the signal is 3.11 × 10^−2^ V. It can be seen that the CO_2_ concentration in the detection structure showed almost no significant change within a short time period.

### 4.6 Measurement process of photosynthetic rate in plant leaves

**Fig. 11:**
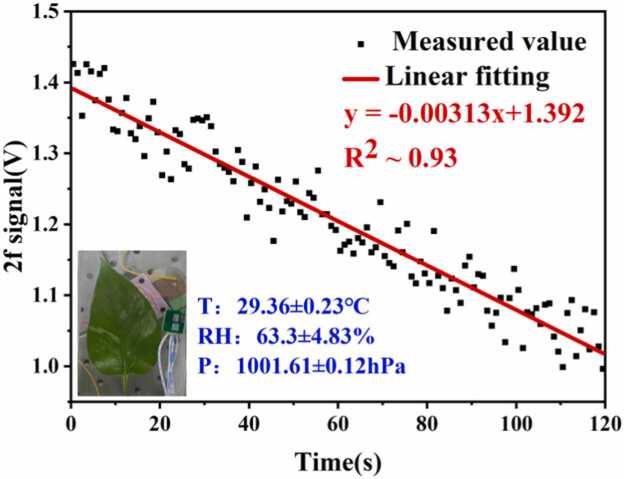
Leaf photosynthesis monitoring data.

The gas supply system in Fig. 4 consists of three gas sources: 2000 ppmv CO_2_ standard gas, high-purity O_2_, and N_2_. Each gas source was connected via polyurethane tubing to the MFC, where they were mixed according to a preset ratio to produce simulated air with a CO_2_ volume fraction of 1000 ppmv. To prevent dry gas from reducing stomatal conductance and photosynthetic rates, the mixed gas underwent humidity adjustment through a gas-washing bottle (containing only a bottom layer of liquid water) before entering the detection structure. Following the above method, after the detection structure forms a sealed chamber, the LED light source was activated to illuminate the leaf. The analog-to-digital converter (ADC, Beijing Xingshuo Huachuang Technology Co., Ltd., FCFR-USB7020) then captured the peak amplitude of the 2 f signal under frequency-locked state in real time. The system's integration time was set to 1 s. The Epipremnum aureum leaf was placed in the leaf chamber to initiate the CO_2_ monitoring experiment. After 120 s of continuous monitoring, a significant change in the 2 f signal amplitude was observed, concluding the monitoring of the Epipremnum aureum leaf's photosynthetic rate. As shown in Fig. 11, the 2 f signal amplitude gradually decreased as the CO_2_ absorbed by the leaf through photosynthesis increased. These results confirm the effectiveness of QEPAS in rapidly monitoring CO_2_ changes during plant photosynthesis.

Eq. (6) quantifies the relationship between CO_2_ concentration change due to photosynthesis under closed-chamber conditions and time, chamber volume, temperature, pressure, and leaf area:

(6) $$P_{n}=\frac{P\cdot V_{cell}}{RT}\cdot \frac{\left(C_{2}-C_{1}\right)}{t_{2}-t_{1}}\cdot \frac{1}{Aleaf}$$

where *P*_*n*_ is the net photosynthetic rate (μmol CO_2_·m^−2^·s^−1^), *P* is the atmospheric pressure (Pa), R is the universal gas constant (8.314 Pa·m^3^·mol^−1^·K^−1^), *T* is the absolute temperature (K), *C*_*2*_*-C*_*1*_ represents the CO_2_ concentration difference (μmol·mol^−1^) over the time interval *t*_*2*_*-t*_*1*_ (s), *V*_*cell*_ is the detection structure volume (m^3^), and *A*_*leaf*_ is the leaf area (m^2^).

As shown in Fig. 11, a linear fit was applied to the actual measured data points within the 120-second measurement period. The linear fit yielded a sensitivity of 3.13 × 10^−3^ V and an R^2^ value of 0.93. The variation in the amplitude of the 2 f signal can be converted into changes in CO_2_ concentration via the linear function shown in Fig. 8. After conversion, the concentration difference *C*_*2*_*-C*_*1*_ in Fig. 11 is 270.24 ppmv, and the time difference *t*_*2*_*-t*_*1*_ is 120 s. The temperature-humidity- pressure sensor (T/H/P sensor) indicated that during the measurement process, *T* was 29.36 ± 0.23 °C and *P* was 1001.61 ± 0.12 hPa. The leaf area was calculated using the grid method, yielding *A*_*leaf*_ = 33 cm^2^. The volume *V*_*cell*_ of the detection structure was determined to be 113.67 cm^3^. Using Eq. (6), the single measurement result of the leaf photosynthetic rate *P*_*n*_ in Fig. 11 is calculated as 3.045 μmol CO_2_·m^−2^·s^−1^.

## 5 Results and discussions

### 5.1 Measurement of leaf photosynthesis

**Fig. 12:**
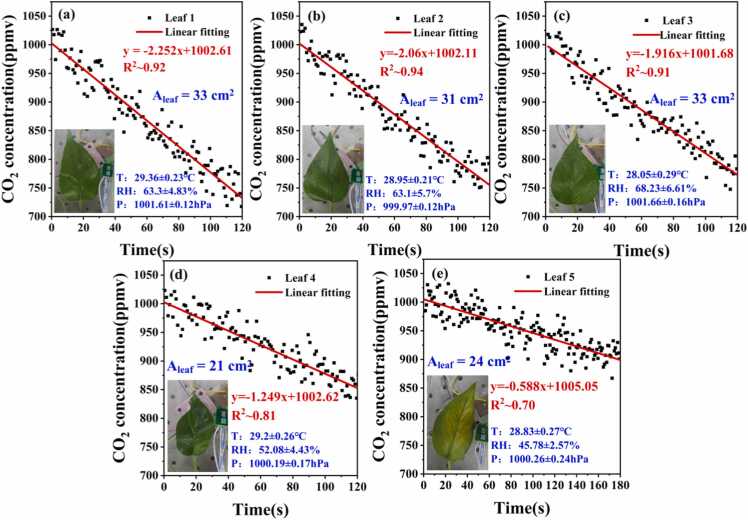
CO₂ depletion curves measured for five individual leaves under identical photosynthetic conditions, with linear-regression fits shown. A_leaf_ denotes the individual leaf area, and T/RH/P indicates the air temperature, relative humidity, and atmospheric pressure. Insets display the morphology and physiological status of each corresponding leaf.

The experiment selected the common indoor plant, Epipremnum aureum, as the research subject to validate the feasibility of QEPAS combined with 1f-locking technology in measuring photosynthesis. During the test, three healthy leaves with well-developed growth (Leaf 1, 2, and 3), one smaller leaf (Leaf 4), and one leaf located on the shaded side showing signs of aging (Leaf 5) were selected for comparative measurements. For leaves 1–4, the single measurement time was set to 120 s. For leaf 5, due to its reduced chlorophyll content and relatively weaker photosynthetic rate [53], the measurement time was extended to 180 s to obtain a clearer CO_2_ absorption change signal. Each leaf was measured five times. The 2 f signal was converted to CO_2_ concentration via the linear function shown in Fig. 8. By performing linear fitting on the concentration variation segment, the corresponding linear function and R^2^ value were obtained. The results from the one experiments for the five kinds of leaves are presented in Fig. 12. The content displayed in the figure includes the original measurement data points, the results of linear fitting, the photograph of the measured leaf, and the ranges of temperature, humidity, and pressure variations monitored by the T/H/P sensor.

### 5.2 Calculation of photosynthetic rate

**Fig. 13:**
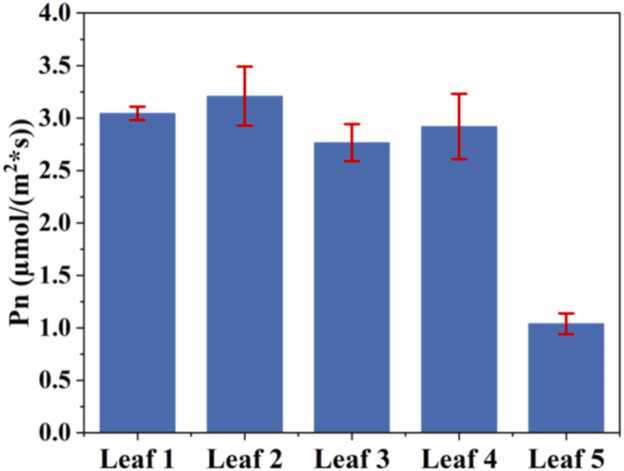
The net photosynthetic rate of five leaves.

Fig. 13 displays the average photosynthetic rates obtained from five replicate measurements for each of the five leaves. Overall, the average photosynthetic rates for leaves 1–4 (large and small leaf types) fluctuated around 3 μmol·m^−2^·s^−1^, indicating that these leaves possess comparable CO_2_ fixation capacities per unit area. Although numerous studies have reported the photosynthetic rates of Epipremnum aureum, measurements vary across experiments due to individual plant differences and differing environmental conditions. Nevertheless, most published data indicates that the photosynthetic rate of indoor Epipremnum aureum typically ranges from 1 to 5.5 μmol·m^−2^·s^−1^ [54], [55], [56]. Therefore, the results obtained in this study fall within a physiologically reasonable range supported by the literature. Additionally, the photosynthetic rate of leaf 5 (the senescent leaf) is markedly reduced, reaching only approximately 1 μmol·m^−2^·s^−1^, exhibiting significant physiological decline traits. The above results demonstrate that the photosynthesis measurement method based on QEPAS combined with 1f-locking technology exhibits excellent reliability and feasibility.

## 6 Conclusion

This paper proposes a method for rapid measurement of plant photosynthetic rates based on QEPAS with 1f-locking technology. A compact gas sensor structure was constructed by integrating an in-plane acoustic micro-resonator (AmR) with a commercial 30.720 kHz quartz tuning fork (QTF). The DFB laser’s output wavelength was locked to the CO_2_ absorption line at 4991.26 cm^−1^ via 1f-locking, enabling real-time monitoring of CO_2_ uptake during leaf photosynthesis. Compared to the scanning mode, the STD in 1f-locking mode is significantly reduced, with detection sensitivity increased by nearly threefold. Experimental results indicate that the system demonstrated a response time of 1 s and excellent linear response (R^2^ = 0.999), with an MDL as low as 2.44 ppmv. The stability and reliability of the device were verified through airtightness performance testing. At the measurement duration of 120 s, the photosynthetic rates of leaves 1–4 stabilized at approximately 3 μmol·m^−2^·s^−1^, consistent with the photosynthetic rate range reported in the literature for Epipremnum aureum. This indicates that the system's measurement results are reasonable. The study also examined leaves at different developmental stages, including mature leaves (1−4) and senescent leaf (5). The photosynthetic rate of senescent leaf is only approximately 1 μmol·m^−2^·s^−1^. Results indicate that the developmental state of leaves plays a significant role in determining photosynthetic rates. Given that Epipremnum aureum is a typical shade- tolerant plant with a relatively low photosynthetic rate, applying this method to plant species with higher photosynthetic rates is expected to further reduce detection time. QEPAS with 1f-locking technology, with its potential for high sensitivity, rapid response, and miniaturization, offers new avenues for overcoming the limitations of traditional detection methods. To maintain low cost and simplicity, a low-power DFB laser and a commercial QTF were used in this work, which is sufficient to validate the proposed photosynthesis sensing platform but limits the ultimate sensitivity. Future improvements can be achieved by employing higher-power lasers and more sensitive acoustic transducers, such as custom QTFs or cantilever-based detectors, to further enhance detection performance. Through continuous optimization, this technology is poised to play a pivotal role in deciphering plant microphysiology, monitoring rapid dynamic changes, and enabling high-throughput phenotyping.

## CRediT authorship contribution statement

**Wen Liu:** Supervision. **Juncheng Lu:** Investigation. **Jie Shao:** Writing – review & editing, Funding acquisition. **Surui Liu:** Writing – original draft, Methodology, Investigation, Conceptualization. **Chongqiu Zhou:** Software. **Lu Qin:** Project administration.

## Funding

This study was supported by the 10.13039/501100001809National Natural Science Foundation of China (61775797), Key Research and Development of Zhejiang Province (2022C03066), Key Science and Technology project of Jinhua City (20213032).

## Declaration of Competing Interest

The authors declare that there are no conflicts of interest.

## Data Availability

Data will be made available on request.
